# 4-[(1-Benzyl-1*H*-1,2,3-triazol-4-yl)meth­oxy]benzene-1,2-dicarbo­nitrile: crystal structure, Hirshfeld surface analysis and energy-minimization calculations

**DOI:** 10.1107/S2056989016004722

**Published:** 2016-03-31

**Authors:** Norzianah Shamsudin, Ai Ling Tan, David J. Young, Mukesh M. Jotani, A. Otero-de-la-Roza, Edward R. T. Tiekink

**Affiliations:** a Faculty of Science, Universiti Brunei Darussalam, Jalan Tungku Link BE 1410, Negara Brunei Darussalam; b Faculty of Science, Health, Education and Engineering, University of the Sunshine Coast, Maroochydore DC, Queensland 4558, Australia; c Department of Physics, Bhavan’s Sheth R. A. College of Science, Ahmedabad, Gujarat 380 001, India; d National Institute for Nanotechnology, National Research Council of Canada, 11421 Saskatchewan Drive, Edmonton, Alberta, T6G 2M9, Canada; e Department of Chemistry, University of British Columbia, Okanagan, 3247 University Way, Kelowna, British Columbia, V1V 1V7, Canada; f Research Centre for Crystalline Materials, Faculty of Science and Technology, Sunway University, 47500 Bandar Sunway, Selangor Darul Ehsan, Malaysia

**Keywords:** crystal structure, triazol­yl, conformation, DFT, Hirshfeld surface

## Abstract

In the solid state, the title compound, C_18_H_13_N_5_O, adopts a conformation whereby the phenyl ring and meth­oxy–benzene-1,2-dicarbo­nitrile residue (r.m.s. deviation of the 12 non-H atoms = 0.041 Å) lie to opposite sides of the central triazolyl ring, forming dihedral angles of 79.30 (13) and 64.59 (10)°, respectively; the dihedral angle between the outer rings is 14.88 (9)°. This conformation is nearly 7 kcal mol^−1^ higher in energy than the energy-minimized structure which has a *syn* disposition of the outer rings, enabling intra­molecular π–π inter­actions. In the crystal, methyl­ene-C—H⋯N(triazol­yl) and carbo­nitrile-N⋯π(benzene) inter­actions lead to supra­molecular chains along the *a* axis. Supra­molecular layers in the *ab* plane arise as the chains are connected by benzene-C—H⋯N(carbo­nitrile) inter­actions; layers stack with no directional inter­actions between them. The specified inter­molecular contacts along with other, weaker contributions to the supra­molecular stabilization are analysed in a Hirshfeld surface analysis.

## Abstract 2

The terminal rings in the title compound have an *anti* disposition in contrast to a *syn* conformation calculated in the energy-minimized structure. Supra­molecular layers in the *ab* plane and sustained by methyl­ene-C—H⋯N(triazol­yl) and carbo­nitrile-N⋯π(benzene) inter­actions feature in the mol­ecular packing.

## Chemical context   

We have previously reported the crystal structure of bis­[(phen­yl­methanamine-κN)-(phthalocyaninato-κ^4^ *N*)zinc] phenyl­methanamine tris­olvate (Shamsudin *et al.*, 2015 ▸) for use as a light-harvesting dye in dye-sensitized solar cells (DSSCs) (Kitamura *et al.*, 2004 ▸, Nazeeruddin *et al.*, 2001 ▸). Benzyl­amine was investigated as a solvent to assist coating TiO_2_ nanoparticles with the highly insoluble zinc phthalocyanine. Another strategy for solubilizing phthalocyanine dyes is to append solubilizing groups to these large, aromatic structures (Mack *et al.*, 2006 ▸). Phthalocyanines are somewhat unreactive and so this is most easily done by modifying the precursor phthalo­nitriles. Unsymmetrical phthalocyanines (*e.g*. tetra- rather than octa-substituted) can yield constitutional isomers, but are more soluble (Eberhart & Hanack, 1997 ▸) and have a greater dipole moment which can make attractive mol­ecules for non-linear optical applications (Tian *et al.*, 1997 ▸). A particularly versatile and reliable reaction for the synthesis of analogues is the azide-alkyne Huisgen cyclo­addition – the best known and most widely used reaction in the ‘click chemistry’ stable (Kolb *et al.*, 2001 ▸). We therefore prepared 3-(prop-2-yn-1-yl­oxy)phthalo­nitrile by the S_N_Ar reaction of propagyl alcohol and 4-nitro­phthalo­nitrile (Jan *et al.*, 2013 ▸) and used this as a precursor for the synthesis of the title mol­ecule (I), the structure of which is described herein along with a Hirshfeld surface analysis and the results of energy-minimization calculations.
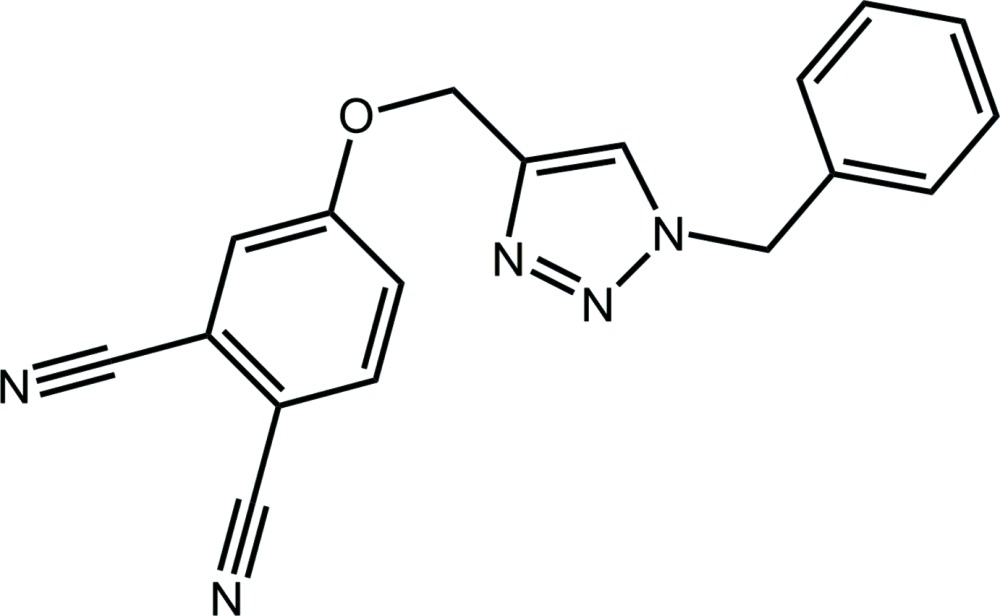


## Structural commentary   

**Figure 1:**
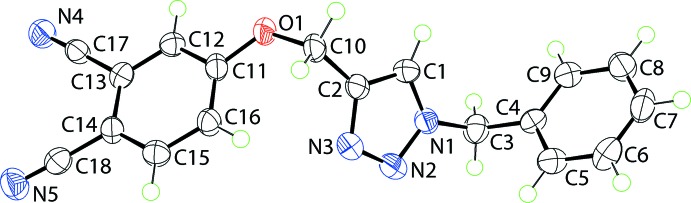
The mol­ecular structure of (I), showing the atom-labelling scheme and displacement ellipsoids at the 70% probability level.

The central five-membered triazolyl ring in (I), Fig. 1 ▸, is strictly planar with the r.m.s. deviation for the five atoms being 0.003 Å. The phenyl ring of the N-bound benzyl group is almost perpendicular to this plane, forming a dihedral angle of 79.30 (13)°. The 12 atoms comprising the meth­oxy–benzene-1,2-dicarbo­nitrile residue are almost co-planar with a r.m.s. deviation of 0.041 Å; the maximum and minimum deviations are −0.085 (2) and 0.038 (2) Å for atoms C10 and C12, respectively. Within the triazolyl ring, the N2—N3 and C1—C2 bond lengths of 1.322 (3) and 1.367 (3) Å, respectively, are consistent with considerable double-bond character in each of these bonds, *i.e*. consistent with the electronic structure shown in the Scheme. The meth­oxy–benzene-1,2-dicarbo­nitrile residue lies to the opposite side of the central ring to the benzyl residue and forms a dihedral angle of 64.59 (10)° with the triazolyl ring. The overall shape of the mol­ecule is thus best described as a step with a dihedral angle between the outer rings of 14.62 (12)°, consistent with these groups being approximately parallel.

## Supra­molecular features   

**Figure 2:**
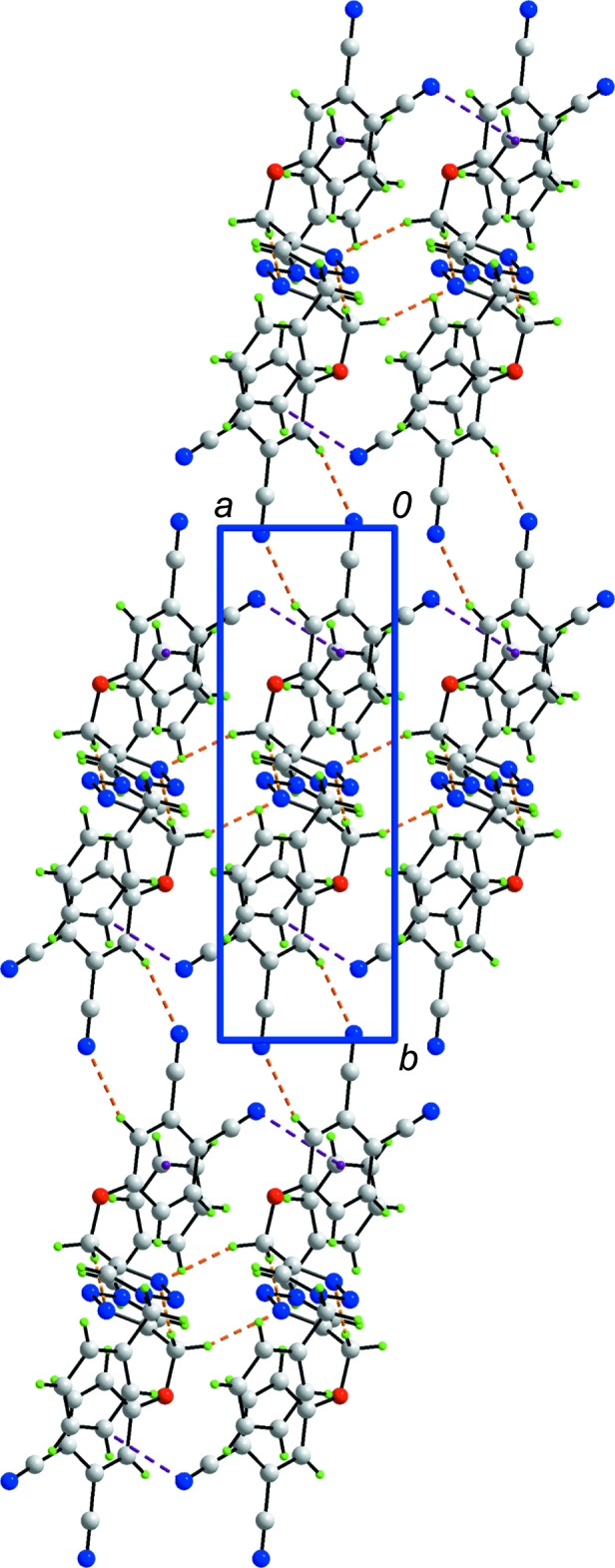
A view of the supra­molecular layer in the *ab* plane in (I). The layer is sustained by C—H⋯N and C—H⋯N inter­actions shown as orange and purple dashed lines, respectively.

**Figure 3:**
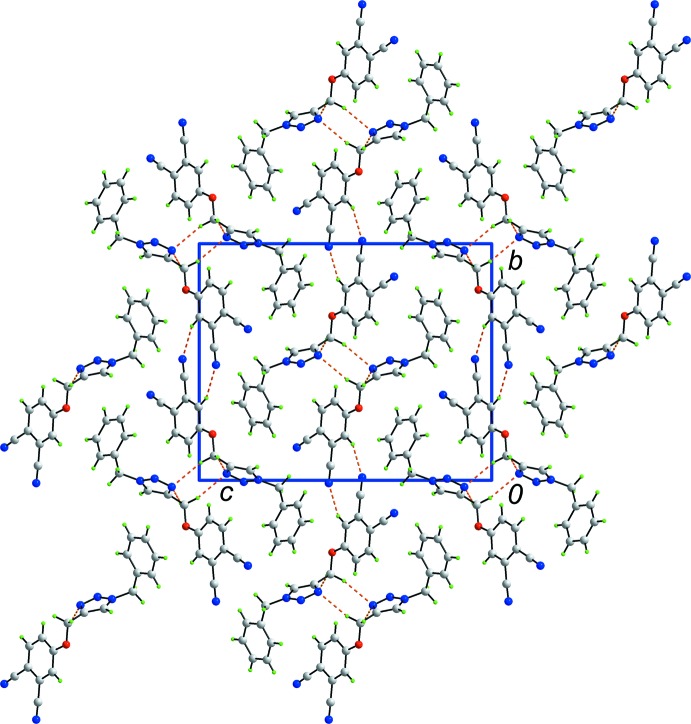
Unit cell contents for (I) shown in projection down the *a* axis, showing the stacking of layers. The C—H⋯N inter­actions are shown as orange dashed lines.

The mol­ecular packing in the crystal leads to supra­molecular chains along the *a* axis, formed through the agency of methyl­ene-C10—H⋯N3(triazol­yl) inter­actions involving both methyl­ene-H atoms, which both link to N3 (Table 1 ▸). Encompassed within the chains are carbo­nitrile-N5⋯π(benzene) inter­actions, Table 1 ▸. The chains are connected into supra­molecular layers in the *ab* plane by benzene-C12—H⋯N4(carbo­nitrile) inter­actions across a centre of inversion so that ten-membered {⋯HC_3_N}_2_ synthons are formed, Fig. 2 ▸ and Table 1 ▸. Layers inter-digitate along the *c* axis but do not form contacts within the standard distance criteria (Spek, 2009 ▸), Fig. 3 ▸.

## Hirshfeld surface analysis   

The program *Crystal Explorer 3.1* (Wolff *et al.*, 2012 ▸) was used to generate Hirshfeld surfaces mapped over *d*_norm_, *d*_e_, curvedness and electrostatic potential. The electrostatic potential was calculated with *TONTO* (Spackman *et al.*, 2008 ▸; Jayatilaka *et al.*, 2005 ▸), integrated in *Crystal Explorer*, using the experimental geometry as the input. The electrostatic potentials were mapped on the Hirshfeld surface using the STO-3G basis set at the Hartree–Fock level of theory over a range ±0.075 au. The contact distances *d*_i_ and *d*_e_ from the Hirshfeld surface to the nearest atom inside and outside, respectively, enables the analysis of the inter­molecular inter­actions through the mapping of *d*_norm_. The combination of *d*_e_ and *d*_i_ in the form of a two-dimensional fingerprint plot (Rohl *et al.*, 2008 ▸) provides a summary of the inter­molecular contacts in the crystal.

**Figure 4:**
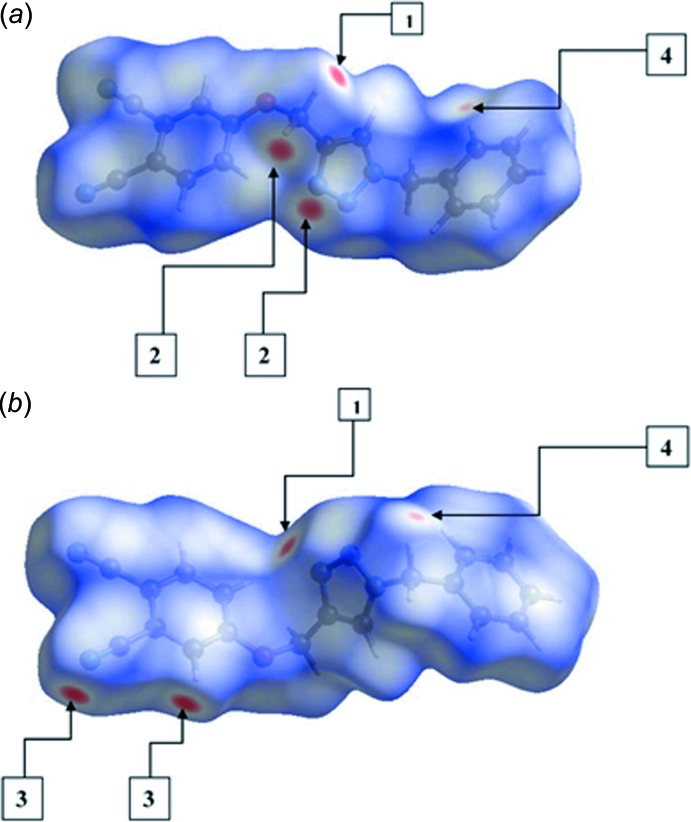
Two views of the Hirshfeld surfaces for (I) mapped over *d*_norm_.

**Figure 5:**
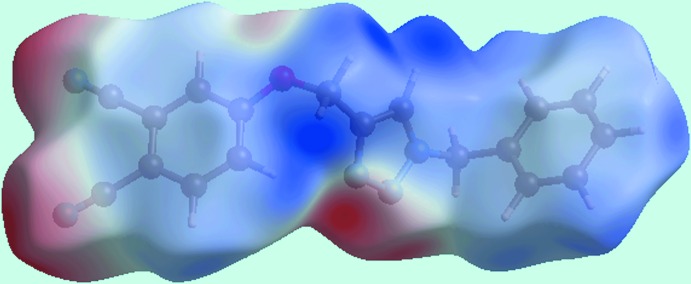
Hirshfeld surface for (I) mapped over the electrostatic potential.

**Figure 6:**
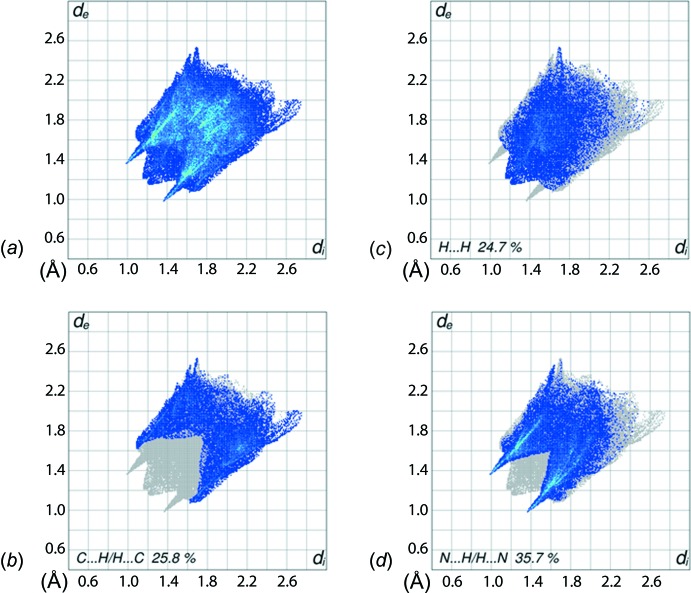
The two-dimensional fingerprint plots for (I): (*a*) all inter­actions, and delineated into (*b*) H⋯H, (*c*) C⋯H/H⋯C, and (*d*) N⋯H/H⋯N inter­actions.

The inter­molecular inter­actions of the C—H⋯N type involving triazolyl-N3 and carbo­nitrile-N4 atoms as hydrogen-bond acceptors, and the H10*A*, H10*B* and H12 hydrogen atoms as donors dominate the mol­ecular packing. These inter­actions are easily recognized as bright-red spots, and are designated as 1, 2 and 3 in a square box, respectively, on the Hirshfeld surface mapped with *d*_norm_ in Fig. 4 ▸. The surface mapped with electrostatic potential, Fig. 5 ▸, highlights these inter­actions as blue and red regions corresponding to positive (donor) and negative (acceptor) electrostatic potentials. The presence of such dominating inter­actions are also evident from the two dimensional fingerprint (FP) plots, Fig. 6 ▸; relative contributions to the overall surface are given in Table 2 ▸.

The prominent pair of sharp spikes of equal lengths (*d*_e_ + *d*_i_ ∼ 2.25 Å) in the FP plot delineated into N⋯H/H⋯N contacts, Fig. 6 ▸ *d*, with a significant contribution to the overall Hirshfeld surface, *i.e*. 35.7% from N⋯H/H⋯N contacts, and the distinct pair of wings corresponding to C⋯H/H⋯C contacts, Fig. 6 ▸ *c*, with a 25.8% contribution, combined, have a greater effect on the mol­ecular packing than the dispersive H⋯H contacts, Fig. 6 ▸ *b*. The diminutive red spots on the surface mapped with *d*_norm_, designated as 4 in a square box of Fig. 4 ▸, at the phenyl-C9 and methyl­ene-H3*B* atoms, reflect the presence of short inter­molecular C⋯H contacts [C9⋯H3*B* ^i^ = 2.79 Å for symmetry code: (i) −1 + *x*, *y*, *z*]. The short intra­molecular H⋯H contact between the benzene-H16 and O-methyl­ene-H10*A* atoms (H10*A*⋯H16 = 2.09 Å) can be recognized from two neighbouring blue regions on the surface mapped with electrostatic potential in Fig. 5 ▸.

**Figure 7:**
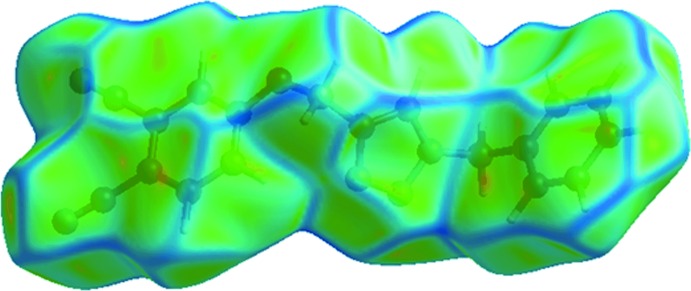
Hirshfeld surface for (I) mapped over curvedness.

The presence of a comparatively weak C—N⋯π inter­action can be viewed from the negative potential around the carbo­nitrile-N5 atom (red region) and the light-blue region around the phenyl ring in Fig. 5 ▸; the strength of this inter­action is qu­anti­fied as 3.7 and 3.5% relative contribution from C⋯C and C⋯N contacts to the surface. The small flat segments delineated by a blue outline in the surface mapped with curvedness, Fig. 7 ▸, and the small contribution from C⋯C contacts, *i.e*. 3.5%, to the surface is consistent with the absence of significant π–π stacking inter­actions in the structure.

## Database survey   

**Figure 8:**
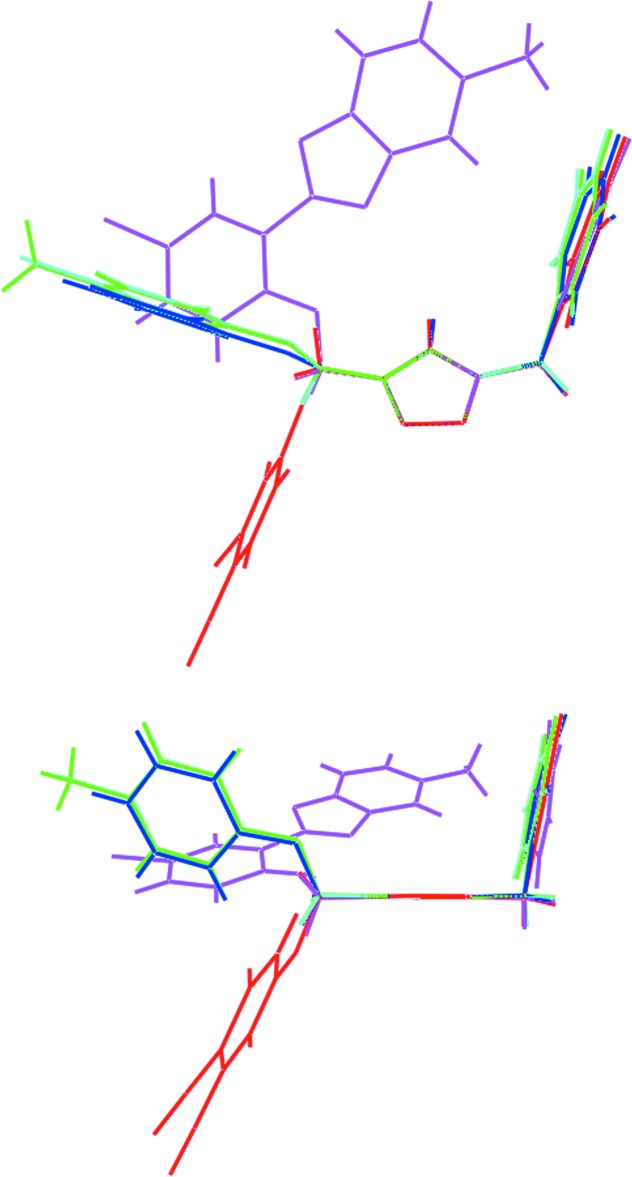
Two views of the different conformations in (I) red image, (II) blue, (III) green, (IV) aqua and (V) pink. The mol­ecules have been overlapped so that the central rings are coincident.

There are four closely related structures to (I) in the crystallographic literature (Groom & Allen, 2014 ▸). The chemical structures of (II)–(V) are shown in Scheme 2, salient dihedral angles are given in Table 3 ▸ and a comparison between mol­ecules is shown in Fig. 8 ▸. The similarity in the structures is seen in the relationship between the central triazolyl ring and pendent phenyl rings. By contrast to the conformation observed in (I), which was described above as *anti* with respect to the relative orientation of the N- and C-bound residues to the central ring, a *syn* disposition is observed in each of (II) (Rostovtsev *et al.*, 2002 ▸), (III) (Garcia *et al.*, 2011 ▸) and (IV) (López-Ruiz *et al.*, 2013 ▸). A similar but somewhat flattened *syn* relationship is observed in (V) (López-Ruiz *et al.*, 2013 ▸) for which an intra­molecular O⋯N contact of 2.745 (3) Å is noted between the ether-O and benzoxazole-N atoms. The difference in structures prompted energy-minimization calculations.
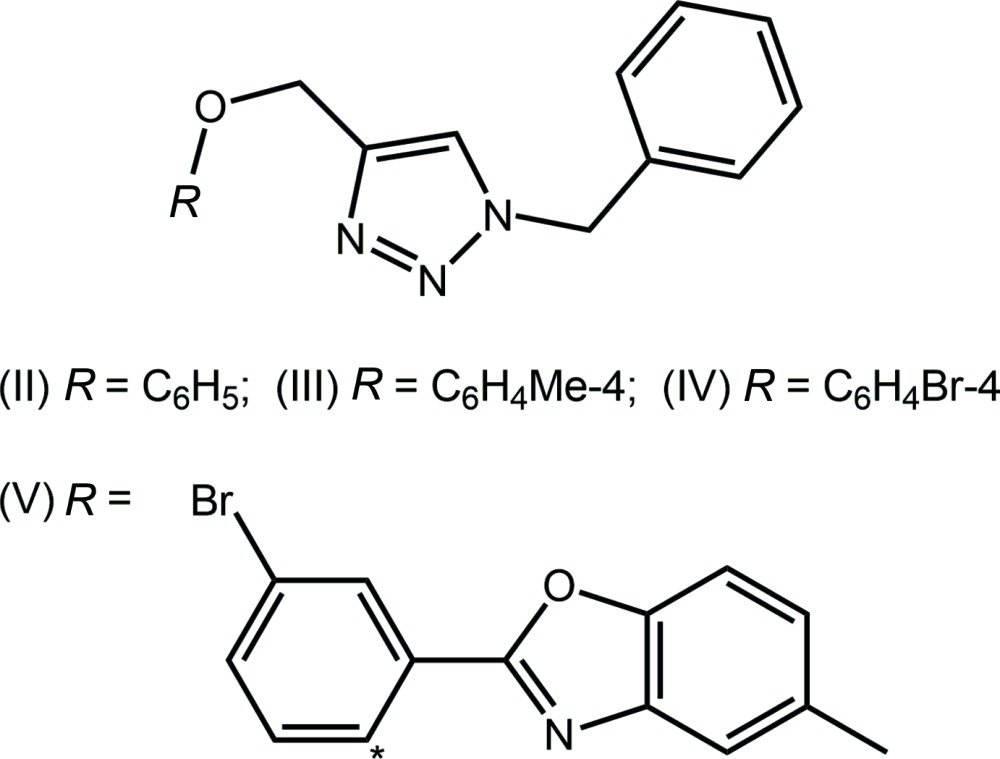


## Energy-minimization calculations   

**Figure 9:**
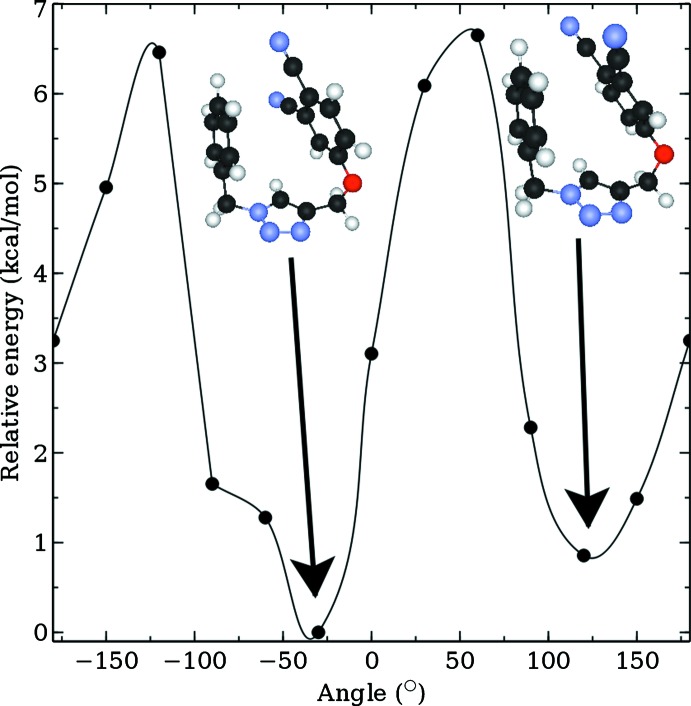
Energy profile (kcal mol^−1^) for conformations of (I) differing by a rotation (30° steps) about the O—C bond.

**Figure 10:**
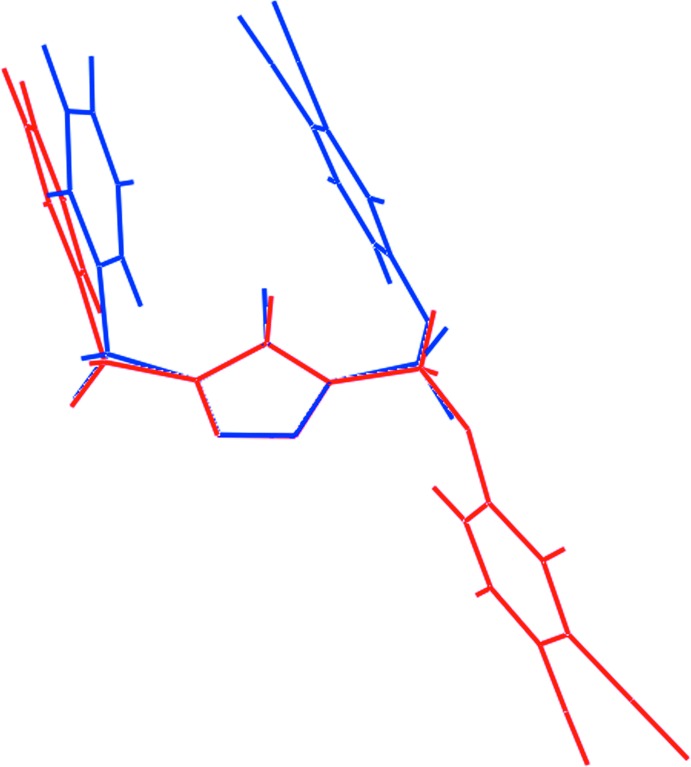
Overlay diagram of the experimental (red image) and energy-minimized (blue) structures of (I). The mol­ecules have been overlapped so that the five-membered rings are coincident.

The structure of (I) was subjected to energy-minimization calculations with Density-Functional Theory (DFT) using the LC-wPBE functional (Vydrov & Scuseria, 2006 ▸; Vydrov *et al.*, 2006 ▸), as implemented in the *Gaussian* program (Frisch *et al.*, 2009 ▸), and the exchange-hole dipole moment (XDM) dispersion correction (Becke & Johnson, 2007 ▸; Otero-de-la-Roza & Johnson, 2013 ▸) with the 6-31+G* basis set. Fig. 9 ▸ shows an energy profile as the 1,2-dicarbo­nitrile residue is rotated (30° steps) about the O—C bond with respect to the rest of the mol­ecule. The energy profile shown in Fig. 9 ▸ reveals the observed *anti* conformation of (I) is in fact a high-energy conformation, being nearly 7 kcal mol^−1^ higher in energy than the low-energy conformation which, as shown in Fig. 10 ▸, has a *syn* conformation of the aromatic rings. In the energy-minimized structure, the dihedral angles between the five-membered ring and the di­nitrile- and benzyl-benzene rings are 73.6 and 85.2°, respectively, *i.e*. differing by *ca* 9 and 6°, respectively, from the comparable angles in the experimental structure. The dihedral angles between the aromatic rings is 23.4°. While the dihedral angles do not differ significantly between the experimental and gas-phase, energy-minimized structures, the relative conformations are quite distinct. The *syn* orientation of the terminal rings is most likely stabilized by intra­molecular π–π inter­actions, the shortest intra­molecular C⋯C contact between rings being 3.62 Å. The adoption of a different conformation in the experimental structure no doubt relates to the dictates of global crystal packing considerations.

## Synthesis and crystallization   

3-(Prop-2-yn-1-yl­oxy)phthalo­nitrile (Jan *et al.*, 2013 ▸; 0.10 g, 0.55 mmol), CuSO_4_ (0.032 g), sodium ascorbate (0.13 g) and benzyl azide (0.074 g) were dissolved in 75% aqueous acetone (20 ml) and stirred for 48 h at room temperature. The reaction was poured into ice–water and the resulting off-white solid was collected by vacuum filtration and was recrystallized as light-brown prisms from a solvent mixture of di­chloro­methane and hexane (0.082 g, 47.5%). M.p.: 397–399 K. IR (ν) 3200 *m* (ArH), 3050 *m* (ArH), 2226 *m* (C≡N), 1600 *s* (C=C, Ar). [*M*+^.^] *m*/*z* 315.

## Refinement details   

Crystal data, data collection and structure refinement details are summarized in Table 4 ▸. Carbon-bound H atoms were placed in calculated positions (C—H = 0.95–0.99 Å) and were included in the refinement in the riding model approximation, with *U*_iso_(H) set to 1.2*U*_eq_(C).

## Supplementary Material

Crystal structure: contains datablock(s) I, global. DOI: 10.1107/S2056989016004722/hb7573sup1.cif

Structure factors: contains datablock(s) I. DOI: 10.1107/S2056989016004722/hb7573Isup2.hkl

Supporting information file. DOI: 10.1107/S2056989016004722/hb7573Isup3.cml

CCDC reference: 1469592

Additional supporting information:  crystallographic information; 3D view; checkCIF report

## Figures and Tables

**Table 1:** Hydrogen-bond geometry (Å, °) *Cg*1 is the centroid of the C11–C16 ring.

*D*—H⋯*A*	*D*—H	H⋯*A*	*D*⋯*A*	*D*—H⋯*A*
C10—H10*A*⋯N3^i^	0.99	2.50	3.468 (3)	167
C10—H10*B*⋯N3^ii^	0.99	2.53	3.477 (3)	161
C12—H12⋯N4^iii^	0.95	2.47	3.353 (3)	155
C18—N5⋯*Cg*1^iv^	1.15 (1)	3.81 (1)	3.853 (2)	83 (1)

Symmetry codes: (i) 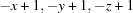; (ii) 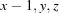; (iii) 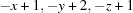; (iv) 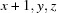.

**Table 2:** Percentage contribution of the different inter­molecular inter­actions to the Hirshfeld surface of (I)

Contact	%
H⋯H	24.7
N⋯H/H⋯N	35.7
C⋯H/H⋯C	25.8
C⋯C	3.7
C⋯N	3.5
O⋯H/H⋯O	3.2
C⋯O	2.7
N⋯N	0.7

**Table 3:** Dihedral angle (°) data for (I) and related literature structures^*a*^

Structure	Triazol­yl/benz­yl-phen­yl	Triazol­yl/*O*-benzene	Benzyl-phen­yl/*O*-benzene	CSD refcode^*b*^	Reference
(I)	79.30 (13)	64.59 (10)	14.88 (9)	–	This work
(II)	77.89 (6)	56.69 (4)	85.82 (5)	CAKSAJ	Rostovtsev *et al.* (2002 ▸)
(III)	79.63 (5)	59.36 95)	85.56 (6)	BEDREJ	Garcia *et al.* (2011 ▸)
(IV)	79.16 (10)	59.57 (11)	84.25 (10)	CIGRUH	López-Ruiz *et al.* (2013 ▸)
(V)	82.03 (9)	26.57 (9)	83.63 (8)	CIGRER	López-Ruiz *et al.* (2013 ▸)

Notes: (*a*) See Scheme 2 for chemical structures; (*b*) Groom & Allen (2014 ▸).

**Table 4:** Experimental details

Crystal data	
Chemical formula	C_18_H_13_N_5_O
*M*_r_	315.33
Crystal system, space group	Monoclinic, *P*2_1_/*c*
Temperature (K)	100
*a*, *b*, *c* (Å)	5.2454 (5), 15.3860 (14), 19.042 (3)
β (°)	90.927 (10)
*V* (Å^3^)	1536.6 (3)
*Z*	4
Radiation type	Mo *K*α
μ (mm^−1^)	0.09
Crystal size (mm)	0.35 × 0.10 × 0.10

Data collection	
Diffractometer	Agilent Technologies SuperNova Dual diffractometer with an Atlas detector
Absorption correction	Multi-scan (*CrysAlis PRO*; Agilent, 2012 ▸)
*T*_min_, *T*_max_	0.588, 1.000
No. of measured, independent and observed [*I* > 2σ(*I*)] reflections	15856, 3527, 2099
*R*_int_	0.080
(sin θ/λ)_max_ (Å^−1^)	0.650

Refinement	
*R*[*F* ^2^ > 2σ(*F* ^2^)], *wR*(*F* ^2^), *S*	0.057, 0.136, 1.07
No. of reflections	3527
No. of parameters	217
H-atom treatment	H-atom parameters constrained
Δρ_max_, Δρ_min_ (e Å^−3^)	0.26, −0.25

Computer programs: *CrysAlis PRO* (Agilent, 2012 ▸), *SHELXS97* (Sheldrick, 2008 ▸), *SHELXL2014* (Sheldrick, 2015 ▸), *ORTEP-3 for Windows* (Farrugia, 2012 ▸), *QMol* (Gans & Shalloway, 2001 ▸), *DIAMOND* (Brandenburg, 2006 ▸) and *publCIF* (Westrip, 2010 ▸).

## supplementary crystallographic information

### Crystal data

**Table:**

C_18_H_13_N_5_O	*F*(000) = 656
*M**_r_* = 315.33	*D*_x_ = 1.363 Mg m^−^^3^
Monoclinic, *P*2_1_/*c*	Mo *K*α radiation, λ = 0.71073 Å
*a* = 5.2454 (5) Å	Cell parameters from 1806 reflections
*b* = 15.3860 (14) Å	θ = 2.5–27.5°
*c* = 19.042 (3) Å	µ = 0.09 mm^−^^1^
β = 90.927 (10)°	*T* = 100 K
*V* = 1536.6 (3) Å^3^	Prism, light-brown
*Z* = 4	0.35 × 0.10 × 0.10 mm

### Data collection

**Table:**

Agilent Technologies SuperNova Dual diffractometer with an Atlas detector	3527 independent reflections
Radiation source: SuperNova (Mo) X-ray Source	2099 reflections with *I* > 2σ(*I*)
Mirror monochromator	*R*_int_ = 0.080
Detector resolution: 10.4041 pixels mm^-1^	θ_max_ = 27.5°, θ_min_ = 2.5°
ω scan	*h* = −6→6
Absorption correction: multi-scan (*CrysAlis PRO*; Agilent, 2012)	*k* = −19→19
*T*_min_ = 0.588, *T*_max_ = 1.000	*l* = −24→21
15856 measured reflections	

### Refinement

**Table:**

Refinement on *F*^2^	0 restraints
Least-squares matrix: full	Hydrogen site location: inferred from neighbouring sites
*R*[*F*^2^ > 2σ(*F*^2^)] = 0.057	H-atom parameters constrained
*wR*(*F*^2^) = 0.136	*w* = 1/[σ^2^(*F*_o_^2^) + (0.0342*P*)^2^ + 0.5378*P*] where *P* = (*F*_o_^2^ + 2*F*_c_^2^)/3
*S* = 1.07	(Δ/σ)_max_ < 0.001
3527 reflections	Δρ_max_ = 0.26 e Å^−^^3^
217 parameters	Δρ_min_ = −0.25 e Å^−^^3^

### Special details

**Table:**

Geometry. All esds (except the esd in the dihedral angle between two l.s. planes) are estimated using the full covariance matrix. The cell esds are taken into account individually in the estimation of esds in distances, angles and torsion angles; correlations between esds in cell parameters are only used when they are defined by crystal symmetry. An approximate (isotropic) treatment of cell esds is used for estimating esds involving l.s. planes.

### Fractional atomic coordinates and isotropic or equivalent isotropic displacement parameters (Å^2^)

**Table:**

	*x*	*y*	*z*	*U*_iso_*/*U*_eq_	
O1	0.3153 (3)	0.69488 (10)	0.54542 (9)	0.0262 (4)	
N1	0.5641 (4)	0.50248 (12)	0.70174 (11)	0.0233 (5)	
N2	0.7432 (4)	0.49329 (13)	0.65250 (12)	0.0294 (5)	
N3	0.6509 (4)	0.52816 (13)	0.59396 (11)	0.0279 (5)	
N4	0.7655 (4)	1.01212 (13)	0.44279 (12)	0.0312 (5)	
N5	1.2066 (4)	0.86108 (14)	0.33358 (13)	0.0342 (6)	
C1	0.3561 (4)	0.54262 (15)	0.67500 (14)	0.0251 (6)	
H1	0.2032	0.5564	0.6987	0.030*	
C2	0.4125 (4)	0.55919 (14)	0.60643 (13)	0.0216 (5)	
C3	0.6072 (5)	0.46954 (16)	0.77327 (14)	0.0282 (6)	
H3A	0.5691	0.5162	0.8073	0.034*	
H3B	0.7892	0.4537	0.7794	0.034*	
C4	0.4444 (4)	0.39128 (15)	0.78895 (13)	0.0234 (5)	
C5	0.4866 (4)	0.31288 (15)	0.75402 (14)	0.0294 (6)	
H5	0.6186	0.3087	0.7206	0.035*	
C6	0.3363 (5)	0.24131 (16)	0.76800 (15)	0.0330 (7)	
H6	0.3662	0.1880	0.7444	0.040*	
C7	0.1427 (5)	0.24702 (17)	0.81622 (15)	0.0329 (7)	
H7	0.0388	0.1979	0.8254	0.040*	
C8	0.1008 (5)	0.32439 (16)	0.85111 (14)	0.0312 (6)	
H8	−0.0313	0.3282	0.8845	0.037*	
C9	0.2511 (4)	0.39631 (16)	0.83744 (14)	0.0273 (6)	
H9	0.2213	0.4494	0.8615	0.033*	
C10	0.2539 (4)	0.60336 (14)	0.55118 (13)	0.0240 (6)	
H10A	0.2818	0.5747	0.5054	0.029*	
H10B	0.0714	0.5970	0.5626	0.029*	
C11	0.5055 (4)	0.72062 (15)	0.50218 (13)	0.0224 (5)	
C12	0.5349 (4)	0.81069 (15)	0.49807 (13)	0.0227 (5)	
H12	0.4294	0.8480	0.5248	0.027*	
C13	0.7180 (4)	0.84514 (14)	0.45502 (13)	0.0224 (5)	
C14	0.8748 (4)	0.79094 (15)	0.41502 (13)	0.0227 (5)	
C15	0.8425 (4)	0.70107 (15)	0.41989 (13)	0.0254 (6)	
H15	0.9464	0.6635	0.3929	0.031*	
C16	0.6609 (4)	0.66612 (15)	0.46354 (13)	0.0236 (5)	
H16	0.6425	0.6049	0.4671	0.028*	
C17	0.7433 (4)	0.93813 (16)	0.44910 (13)	0.0240 (6)	
C18	1.0608 (4)	0.82840 (15)	0.36943 (14)	0.0256 (6)	

### Atomic displacement parameters (Å^2^)

**Table:**

	*U*^11^	*U*^22^	*U*^33^	*U*^12^	*U*^13^	*U*^23^
O1	0.0306 (9)	0.0188 (9)	0.0296 (11)	0.0005 (7)	0.0083 (8)	0.0045 (8)
N1	0.0236 (10)	0.0228 (11)	0.0236 (12)	−0.0017 (8)	0.0033 (9)	0.0012 (9)
N2	0.0230 (10)	0.0349 (12)	0.0305 (14)	0.0018 (9)	0.0057 (10)	0.0039 (10)
N3	0.0262 (11)	0.0301 (12)	0.0276 (13)	0.0001 (9)	0.0053 (9)	0.0031 (10)
N4	0.0359 (12)	0.0237 (12)	0.0344 (14)	0.0005 (9)	0.0068 (10)	0.0009 (10)
N5	0.0384 (12)	0.0288 (12)	0.0358 (15)	−0.0041 (10)	0.0103 (11)	0.0015 (11)
C1	0.0237 (12)	0.0227 (13)	0.0291 (16)	0.0034 (10)	0.0055 (11)	−0.0006 (11)
C2	0.0211 (11)	0.0152 (12)	0.0285 (15)	−0.0037 (9)	0.0044 (10)	−0.0014 (10)
C3	0.0300 (13)	0.0289 (14)	0.0256 (15)	−0.0009 (10)	−0.0024 (11)	0.0036 (12)
C4	0.0228 (12)	0.0229 (13)	0.0245 (15)	0.0035 (10)	−0.0023 (10)	0.0018 (11)
C5	0.0282 (13)	0.0287 (14)	0.0314 (16)	0.0054 (10)	0.0033 (11)	0.0000 (12)
C6	0.0425 (15)	0.0220 (14)	0.0344 (17)	0.0023 (11)	−0.0022 (13)	0.0018 (12)
C7	0.0365 (14)	0.0250 (14)	0.0371 (18)	−0.0042 (11)	−0.0027 (13)	0.0105 (12)
C8	0.0274 (13)	0.0352 (16)	0.0310 (16)	−0.0002 (11)	0.0017 (11)	0.0055 (13)
C9	0.0268 (12)	0.0261 (13)	0.0291 (16)	0.0032 (10)	0.0015 (11)	0.0002 (12)
C10	0.0266 (12)	0.0192 (12)	0.0265 (15)	−0.0036 (9)	0.0057 (11)	0.0030 (11)
C11	0.0215 (11)	0.0247 (13)	0.0211 (14)	−0.0030 (9)	−0.0007 (10)	0.0056 (11)
C12	0.0239 (12)	0.0210 (12)	0.0233 (14)	0.0024 (9)	0.0017 (10)	−0.0007 (10)
C13	0.0254 (12)	0.0193 (12)	0.0224 (14)	−0.0001 (9)	−0.0014 (10)	0.0007 (10)
C14	0.0256 (12)	0.0215 (13)	0.0209 (14)	−0.0014 (10)	0.0019 (10)	0.0023 (10)
C15	0.0279 (12)	0.0239 (13)	0.0247 (15)	0.0021 (10)	0.0039 (11)	−0.0021 (11)
C16	0.0293 (12)	0.0181 (12)	0.0237 (15)	−0.0010 (10)	0.0052 (11)	0.0002 (11)
C17	0.0223 (12)	0.0279 (14)	0.0219 (14)	0.0011 (10)	0.0040 (10)	0.0000 (11)
C18	0.0279 (13)	0.0217 (13)	0.0272 (16)	0.0014 (10)	−0.0004 (12)	−0.0006 (11)

### Geometric parameters (Å, º)

**Table:**

O1—C11	1.363 (3)	C6—H6	0.9500
O1—C10	1.449 (3)	C7—C8	1.382 (4)
N1—N2	1.346 (3)	C7—H7	0.9500
N1—C1	1.347 (3)	C8—C9	1.386 (3)
N1—C3	1.467 (3)	C8—H8	0.9500
N2—N3	1.322 (3)	C9—H9	0.9500
N3—C2	1.363 (3)	C10—H10A	0.9900
N4—C17	1.151 (3)	C10—H10B	0.9900
N5—C18	1.149 (3)	C11—C16	1.388 (3)
C1—C2	1.367 (3)	C11—C12	1.397 (3)
C1—H1	0.9500	C12—C13	1.379 (3)
C2—C10	1.494 (3)	C12—H12	0.9500
C3—C4	1.509 (3)	C13—C14	1.404 (3)
C3—H3A	0.9900	C13—C17	1.441 (3)
C3—H3B	0.9900	C14—C15	1.396 (3)
C4—C9	1.385 (3)	C14—C18	1.437 (4)
C4—C5	1.397 (3)	C15—C16	1.383 (3)
C5—C6	1.383 (3)	C15—H15	0.9500
C5—H5	0.9500	C16—H16	0.9500
C6—C7	1.383 (4)		
			
C11—O1—C10	119.61 (18)	C7—C8—H8	119.9
N2—N1—C1	110.8 (2)	C9—C8—H8	119.9
N2—N1—C3	120.75 (19)	C4—C9—C8	120.4 (2)
C1—N1—C3	128.4 (2)	C4—C9—H9	119.8
N3—N2—N1	107.14 (18)	C8—C9—H9	119.8
N2—N3—C2	108.6 (2)	O1—C10—C2	111.92 (17)
N1—C1—C2	105.1 (2)	O1—C10—H10A	109.2
N1—C1—H1	127.5	C2—C10—H10A	109.2
C2—C1—H1	127.5	O1—C10—H10B	109.2
N3—C2—C1	108.3 (2)	C2—C10—H10B	109.2
N3—C2—C10	122.6 (2)	H10A—C10—H10B	107.9
C1—C2—C10	129.1 (2)	O1—C11—C16	125.9 (2)
N1—C3—C4	112.33 (19)	O1—C11—C12	113.9 (2)
N1—C3—H3A	109.1	C16—C11—C12	120.2 (2)
C4—C3—H3A	109.1	C13—C12—C11	119.6 (2)
N1—C3—H3B	109.1	C13—C12—H12	120.2
C4—C3—H3B	109.1	C11—C12—H12	120.2
H3A—C3—H3B	107.9	C12—C13—C14	120.9 (2)
C9—C4—C5	119.3 (2)	C12—C13—C17	119.6 (2)
C9—C4—C3	120.7 (2)	C14—C13—C17	119.4 (2)
C5—C4—C3	120.0 (2)	C15—C14—C13	118.6 (2)
C6—C5—C4	120.1 (3)	C15—C14—C18	121.4 (2)
C6—C5—H5	120.0	C13—C14—C18	119.9 (2)
C4—C5—H5	120.0	C16—C15—C14	120.7 (2)
C5—C6—C7	120.3 (3)	C16—C15—H15	119.7
C5—C6—H6	119.9	C14—C15—H15	119.7
C7—C6—H6	119.9	C15—C16—C11	120.0 (2)
C8—C7—C6	119.9 (2)	C15—C16—H16	120.0
C8—C7—H7	120.1	C11—C16—H16	120.0
C6—C7—H7	120.1	N4—C17—C13	178.4 (3)
C7—C8—C9	120.1 (3)	N5—C18—C14	177.7 (3)
			
C1—N1—N2—N3	0.4 (2)	C7—C8—C9—C4	−0.1 (4)
C3—N1—N2—N3	179.53 (19)	C11—O1—C10—C2	87.8 (2)
N1—N2—N3—C2	−0.2 (2)	N3—C2—C10—O1	−84.1 (3)
N2—N1—C1—C2	−0.5 (3)	C1—C2—C10—O1	95.6 (3)
C3—N1—C1—C2	−179.5 (2)	C10—O1—C11—C16	−3.0 (3)
N2—N3—C2—C1	−0.1 (3)	C10—O1—C11—C12	176.24 (18)
N2—N3—C2—C10	179.62 (19)	O1—C11—C12—C13	−178.81 (19)
N1—C1—C2—N3	0.4 (3)	C16—C11—C12—C13	0.5 (3)
N1—C1—C2—C10	−179.4 (2)	C11—C12—C13—C14	0.2 (3)
N2—N1—C3—C4	−109.4 (2)	C11—C12—C13—C17	178.1 (2)
C1—N1—C3—C4	69.6 (3)	C12—C13—C14—C15	−0.3 (3)
N1—C3—C4—C9	−112.3 (3)	C17—C13—C14—C15	−178.2 (2)
N1—C3—C4—C5	67.2 (3)	C12—C13—C14—C18	178.7 (2)
C9—C4—C5—C6	0.0 (3)	C17—C13—C14—C18	0.8 (3)
C3—C4—C5—C6	−179.5 (2)	C13—C14—C15—C16	−0.4 (3)
C4—C5—C6—C7	0.4 (4)	C18—C14—C15—C16	−179.4 (2)
C5—C6—C7—C8	−0.6 (4)	C14—C15—C16—C11	1.1 (3)
C6—C7—C8—C9	0.5 (4)	O1—C11—C16—C15	178.1 (2)
C5—C4—C9—C8	−0.1 (3)	C12—C11—C16—C15	−1.1 (3)
C3—C4—C9—C8	179.4 (2)		

### Hydrogen-bond geometry (Å, º)

Cg1 is the centroid of the C11–C16 ring.

**Table:**

*D*—H···*A*	*D*—H	H···*A*	*D*···*A*	*D*—H···*A*
C10—H10*A*···N3^i^	0.99	2.50	3.468 (3)	167
C10—H10*B*···N3^ii^	0.99	2.53	3.477 (3)	161
C12—H12···N4^iii^	0.95	2.47	3.353 (3)	155
C18—N5···*Cg*1^iv^	1.15 (1)	3.81 (1)	3.853 (2)	83 (1)

Symmetry codes: (i) −*x*+1, −*y*+1, −*z*+1; (ii) *x*−1, *y*, *z*; (iii) −*x*+1, −*y*+2, −*z*+1; (iv) *x*+1, *y*, *z*.
